# Early relapse prediction after allogeneic hematopoietic stem cell transplantation for acute lymphoblastic leukemia (ALL) using lineage‐specific chimerism analysis

**DOI:** 10.1002/jha2.568

**Published:** 2022-09-19

**Authors:** Hannes Lindahl, Davide Valentini, Sofie Vonlanthen, Mikael Sundin, Andreas T. Björklund, Stephan Mielke, Dan Hauzenberger

**Affiliations:** ^1^ Clinical Immunology and Transfusion Medicine Karolinska University Hospital Stockholm Sweden; ^2^ Department of Clinical Neuroscience Karolinska Institutet Stockholm Sweden; ^3^ Department of Cellular Therapy and Allogeneic Stem Cell Transplantation (CAST) Karolinska University Hospital Stockholm Sweden; ^4^ Pediatric Hematology Immunology and HCT Astrid Lindgren Children's Hospital Karolinska University Hospital Stockholm Sweden; ^5^ Pediatrics CLINTEC Karolinska Institutet Stockholm Sweden; ^6^ Department of Cell Therapy and Allogeneic Stem Cell Transplantation (CAST) Department of Laboratory Medicine (LabMED) Karolinska University Hospital and Institutet Karolinska Comprehensive Cancer Center Stockholm Sweden

**Keywords:** acute leukemia, chimerism analysis, relapse prediction, stem cell transplantation

## Abstract

Relapse is a major cause of treatment failure after hematopoietic stem cell transplantation (HSCT) for acute leukemia. Here, we report a monocentric retrospective study of all HSCTs for B cell acute lymphoblastic leukemia (ALL) performed during the years 2005–2021 (*n* = 138, including 51 children), aiming to identify the optimal use of lineage‐specific recipient‐donor chimerism analysis for prediction of relapse. In adults, relapse was associated with increased recipient chimerism in CD3^+^ bone marrow cells sampled at least 30 days before a relapse. Relapse could be predicted with a sensitivity of 73% and a specificity of 83%. Results were similar for children but with a higher recipient chimerism cutoff. Additionally, adults that had at least one chimerism value <0.12% in CD3^+^ peripheral blood cells within the first 60 days after HSCT had 89% probability of being relapse‐free after 2‐years compared to 64%. Results were similar for children but again necessitating a higher chimerism cutoff. These results suggest that high‐sensitive lineage‐specific chimerism analysis can be used for (1) early ALL relapse prediction by longitudinal chimerism monitoring in CD3^+^ bone marrow cells and (2) relapse risk stratification by analyzing CD3^+^ blood cells early post‐HSCT.

## 1 INTRODUCTION

Acute lymphoblastic leukemia (ALL) is the most common childhood cancer but can occur at any age. The vast majority of adults with ALL will need an allogeneic hematopoietic stem cell transplantation (HSCT) as definitive therapy to control the disease [1]. In contrast, relatively few cases of pediatric ALL are transplanted but of the 20% that relapse after first remission at least half will get an allogeneic HSCT [2]. HSCT is a potentially curative treatment for high‐risk ALL in all age groups but relapse remains a major cause of treatment failure and is associated with high mortality [3, 4]. It is crucial to detect a developing relapse early when immune interventions, such as tapering of immunosuppression or donor lymphocyte infusion (DLI), are more likely to be successful [4, 5].

Monitoring of recipient‐donor chimerism after allogeneic HSCTs has been clinical routine for decades and is primarily used to assess engraftment [6]. Chimerism analysis has also attracted interest for its potential to be used for early relapse detection as a complement to or substitute for disease‐specific markers of minimal residual disease (MRD), which are not always available [7, 8]. However, there are no established guidelines that describe how chimerism analysis may be used to predict relapses and practices vary considerably worldwide [9]. Clinical trials will be required to definitively show if chimerism analysis can be used to predict relapse early enough to increase survival. The design of such trials should be informed by findings from hypothesis‐generating observational studies. Choices include at which timepoints to sample, what sample types and cell types to use, and what cutoffs should motivate action. Moreover, sampling and interpretation may differ depending on patient category, transplantation type, and disease.

We have recently reported an analysis of chimerism results from the 154 HSCTs performed at our center during the years 2015–2020 for acute myeloid leukemia (AML) [10]. Given the sampling frequency performed in clinical practice during these years it did not appear feasible to predict an emerging relapse in time to intervene, likely due to the rapid disease kinetics of AML relapses. At the time of relapse however, the association with elevated chimerism in CD33^+^ cells, the lineage corresponding to the immunophenotype of AML, was very strong. However, we also showed that achieving complete donor chimerism, in this case defined as at least one chimerism result <0.2% in CD33^+^ blood or bone marrow cells during the first 60 days after HSCT, was associated with a relapse‐free disease course. We proposed that early chimerism results could potentially be used for risk stratification. Here, we report retrospective data on all allogeneic HSCTs for B cell ALL (B‐ALL) performed at our center during the years 2005–2021. Our aim was to identify cutoffs for single chimerism results at which actions are motivated to inhibit an emerging relapse and to replicate the association between failure to achieve early complete donor chimerism and subsequent relapse.

## 2 METHODS

### 2.1 Patients and HSCTs

This study was approved by the Swedish Ethical Review Authority and was conducted in accordance with the Declaration of Helsinki. Informed consent was obtained from all included patients. All consecutive HCSTs for B‐ALL performed at Karolinska University Hospital from 2005 until the end of 2021 were included. Clinical data incorporated in the European Society for Blood and Marrow Transplantation registry were used. Patient and transplant characteristics are presented in Table 1. The conditioning regimens varied and have been categorized as myeloablative conditioning and reduced intensity conditioning according to established criteria [11]. Similar data from HSCTs for AML that have been previously reported is included in figure 4 [10].

**TABLE 1:** Pediatric and adult HSCT characteristics

	All	With relapse	No relapse	*p‐*Value
**Pediatric HSCTs**	51	9 (18)	42 (82)	
Age at HSCT in y, median (range)	11 (2–18)	10 (2–16)	11 (2–18)	0.57
Male	35 (69)	5 (56)	30 (71)	0.44
Type of conditioning				
Myeloablative	48 (94)	9 (100)	39 (93)	1
Reduced intensity	3 (6)	0 (0)	3 (7)	
Antithymocyte globulin pretreatment	29 (57)	4 (44)	25 (60)	0.47
Donor type				
Identical sibling/relative	15 (29)	3 (33)	12 (29)	0.97
Haploidentical related	5 (10)	‐	5 (12)	
Matched unrelated	31 (61)	6 (67)	25 (60)	
Stem cell source				
Peripheral blood	9 (18)	1 (11)	8 (19)	1
Bone marrow	37 (73)	7 (78)	30 (71)	
DLI treatment post‐HSCT	3 (6)	0 (0)	3 (6)	1
GCSF treatment post‐HSCT	12 (24)	1 (11)	11 (26)	0.67
All cause mortality	17 (33)	5 (56)	12 (29)	0.14
Days to engraftment, median (range)	21 (12–43)	19 (12–28)	21 (13–43)	0.50
Years of follow‐up, median (range)	10.4 (1.3–16.7)	9.1 (2.2–16.2)	10.8 (1.3–16.7)	0.95
Days to relapse, median (range)	‐	255 (35–785)	‐	‐
**Adult HSCTs**	87	18 (21)	69 (79)	
Age at HSCT in y, median (range)	39 (18–69)	32 (19–66)	39 (18–69)	0.30
Male	54 (78)	10 (56)	44 (64)	0.59
Type of conditioning				
Myeloablative	54 (63)	13 (72)	41 (60)	0.42
Reduced intensity	32 (37)	5 (28)	27 (40)	
Antithymocyte globulin pretreatment	57 (66)	11 (61)	46 (67)	0.78
Donor type				
Identical sibling/relative	26 (30)	7 (39)	19 (28)	0.73
Haploidentical related	4 (5)	‐	4 (6)	
Matched unrelated	57 (66)	11 (61)	46 (67)	
Stem cell source				
Peripheral blood	79 (91)	17 (94)	62 (90)	1
Bone marrow	6 (8)	1 (6)	5 (7)	
DLI treatment post‐HSCT	3 (3)	1 (6)	2 (3)	0.51
GCSF treatment post‐HSCT	12 (14)	2 (11)	10 (14)	1
All cause mortality*	23 (28)	12 (67)	11 (17)	<0.0001
Days to engraftment, median (range)	17 (11–29)	18 (15–28)	17 (11–29)	0.18
Years of follow‐up, median (range)	8.7 (1.0–16.8)	11.7 (2.5–16.8)	8.0 (1.0–15.9)	0.047
Days to relapse, median (range)	‐	266 (56–1757)	‐	‐

*Note*: Unless otherwise indicated, data are shown as *n* (%). *p‐*Values for comparisons of transplants with relapse and without relapse are calculated using the Fisher's exact test for 2 × 2 comparisons and the *χ* ^2^ test for 2 × 3 comparisons. Numerical data were analyzed using the Mann–Whitney *U* test. Number of days until engraftment of neutrophils is indicated.

Abbreviations: DLI, donor lymphocyte infusion; G‐CSF, granulocyte colony stimulating factor; HSCT, hematopoietic stem cell transplantation; NS, not statistically significant. .

### 2.2 Cell separation and chimerism analysis

Cells were routinely separated into the major cell types (CD3^+^, CD19^+^, or CD33^+^ for blood samples and CD3^+^, CD19^+^, CD33^+^, or CD34^+^ for bone marrow samples), and chimerism was analyzed as previously described [10]. The purity of cell separations is not routinely assessed but has been tested during the validation of the methods. The purity for blood samples was generally >95% and for bone marrow samples 70%–90%. Throughout, samples categorized as positive for a CD marker are merely enriched for cells positive for the CD marker. Sampling was performed at each clinician's discretion. Chimerism results are presented as percent recipient throughout.

### 2.3 Statistical methods

Descriptive statistics of patient characteristics and transplantation regimens are reported for adult and pediatric (<18 years) patients separately. Eight patients had their first and second HSCT within the study time making the total number of patients in the study fewer than the total number of transplantations. However, for all statistical models, each transplantation is treated equally. Groups were compared using the Fisher's exact test and *χ*2 test for categorical data with 2 × 2 and 2 × 3 comparisons, respectively, and with the Mann–Whitney *U*‐test for numerical data. Univariable and multivariable logistic regression analyses were performed to assess the relationship between relapse and chimerism values as well as other independent variables that were considered potential confounders. Inclusion of these covariates was not based on significant results in univariable analysis but included for face validity. Receiver operating characteristics (ROC)‐curve analysis was used to determine discriminative performance of the models and the optimal cutoff was determined using the Youden's J statistic (Youden's index). Relapse‐free survival was calculated with the Kaplan–Meier method, and the log‐rank test was used for comparisons of curves. Correlations for numerical data were calculated using Pearson's correlation coefficient. *p*‐Values are two sided and 0.05 was considered statistically significant. Locally estimated scatterplot smoothing was used to fit lines to scatterplots for hypothesis‐generation. This was considered an exploratory study, and *p*‐values are presented without correction for multiple testing. All calculations and figures were performed using R v 3.6.1 (the Comprehensive R Archive Network project) with RStudio v. 1.1.456 and the packages ggplot2, ROCit, and survminer.

## 3 RESULTS

### 3.1 Patients and samples

A total of 138 HSCTs were performed for 130 patients with B‐ALL during 2005–2021. Eight patients were transplanted twice during this time. Of the total number of HSCTs, 51 were for pediatric and 87 for adult patients. The median patient age in the pediatric age group was 11 years (range 2–18) and in the adult age group it was 39 [18‐69]. The median follow‐up time for all patients was 9.3 years (range 1.0–16.8). Twenty‐seven transplants (20%) resulted in a relapse within the study time after a median of 264 days post‐HSCT. No statistically significant association between relapse and age, sex, type of conditioning, donor type, or stem cell source was observed (Table 1). Relapse was however associated with death in adult patients but not in the pediatric patients due to a higher proportion of nonrelapsed pediatric patients that had died at the time of this analysis compared to adults (29% vs. 17%).

**FIGURE 1:**
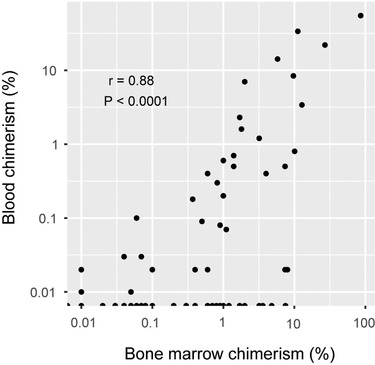
Correlation between blood and bone marrow chimerism. All instances when blood and bone marrow was sampled on the same day from the same patient was extracted. Percent recipient DNA in CD3^+^ cells is plotted. Pearson correlation coefficient r is indicated

All chimerism data for these HSCTs were extracted, in total 5409 results from 1713 unique biological samples (Table 2). Six HSCTs had no associated chimerism results leaving 132 HSCTs for further analysis. Whenever sampling of bone marrow and blood was performed on the same day, these were considered paired samples. The correlation between chimerism values from paired CD3^+^ samples was assessed (Figure 1). For many of these paired samples the values in blood and bone marrow correlated well but instances when bone marrow chimerism values were as high as 1% with negative blood samples drawn the same day were quite frequent, which has been described before [12, 13].

**TABLE 2:** Chimerism data

	All		Pediatric		Adult	
*Bone marrow*	Relapse	No relapse	Relapse	No relapse	Relapse	No relapse
HSCTs with data, *n* (%)	26 (96)	95 (86)	9 (100)	35 (83)	17 (94)	60 (90)
Samples per HSCT	4 (2–7)	4 (3–6)	3 (1–8)	3 (3–4)	4 (3–7)	5 (4–7)
Sampling interval, days	80 (42–146)	‐	98 (35–157)	‐	70 (42–132)	‐
***Blood***						
HSCTs with data, *n* (%)	27 (100)	103 (93)	9 (100)	38 (90)	18 (100)	65 (97)
Samples per HSCT	5 (4–12)	7 (3–12)	8 (4–14)	10 (7–14)	5 (4–9)	5 (2–9)
Sampling interval, days	39 (29–62)	‐	35 (32–45)	‐	49 (28–92)	‐

*Note*: Number of HSCTs with available associated chimerism data. Average sampling interval from HSCT to relapse is indicated. Unless otherwise indicated data are shown as median (IQR, interquartile range).

Abbreviation: HSCT, hematopoietic stem cell transplantation.

### 3.2 Mixed chimerism and prediction of relapse

To assess if single elevated chimerism values can be used to predict a relapse with time to intervene we first filtered the data. As others have done [14], we removed samples that were obtained later than 30 days before a relapse. To adjust the observation time in the two groups (relapse and nonrelapse), we removed values that were obtained in the nonrelapse group later than 30 days before the average time to relapse in the relapse group. Lastly, we removed values that were obtained during the first 30 days after HSCT because mixed chimerism during this time most likely reflects engraftment and not a relapse. After these filtration steps, 225 bone marrow samples from 100 transplants of which 22 led to a relapse and 501 blood samples from 93 transplants of which 21 led to a relapse, were available for analysis.

### 3.3 Prediction of relapse using bone marrow T cell chimerism

**FIGURE 2:**
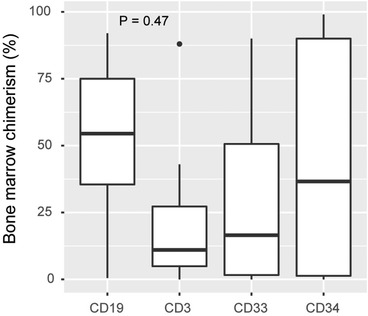
Bone marrow chimerism at relapse. All instances (*n* = 8) when bone marrow was sampled at relapse +/− 3 days. Boxes represent median and interquartile range. Percent recipient DNA is plotted. The *p*‐value was calculated using analysis of variance (ANOVA)

**FIGURE 3:**
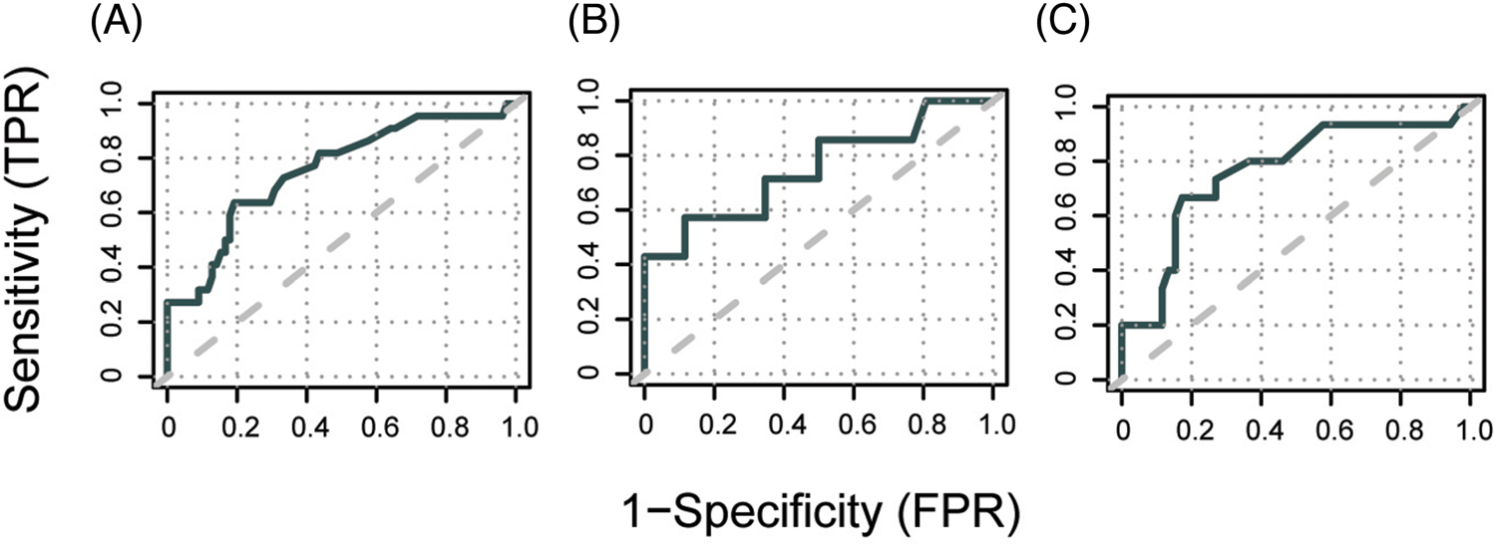
Discriminative performance of single CD3^+^ bone marrow cell chimerism values to predict subsequent relapse. The highest recorded chimerism value in CD3^+^ bone marrow cells from each hematopoietic stem cell transplantation (HSCT) for B‐acute lymphoblastic leukemia (ALL) was used to predict a subsequent relapse. Receiver operating characteristics (ROC)‐curves depict sensitivity and 1‐specificity of relapse prediction in (A) all patients (B) only pediatric patients and (C) only adult patients. Analysis includes chimerism values obtained from 30 days after transplantation to 30 days before clinical relapse or an equal length of time in the patients that did not have a relapse. TPR, true positive rate; FPR, false positive rate

The variable %_max_ was defined for each transplantation as the highest chimerism value that remained after the filtration steps. Univariable logistic regression analysis demonstrated a statistically significant association between relapse and %_max_ in all four bone marrow cell fractions with the largest odds ratio observed when analyzing values from CD3^+^ cells (Table 3). Multivariable analysis showed that this association was independent of other plausible predictive factors of relapse. For comparison, we extracted values obtained at relapse +/− 3 days (*n* = 8, all adult) and observed elevated chimerism values in all four cell subsets in most of the samples (Figure 2). In these samples we observed the lowest median chimerism in the CD3^+^ cells, although the differences in medians for the four subsets were not statistically significant. For %_max_ in bone marrow CD3^+^ cells, ROC‐analysis resulted in an area under the curve (AUC) of 0.75 (Figure 3A). Youden's index suggested an optimal cutoff of 1.6%, which resulted in a sensitivity of 64% and a specificity of 81%. Essentially, this implies that whenever a result of 1.6% or higher in a CD3^+^ bone marrow sample has been obtained the risk of future relapse is elevated. The median time between last bone marrow sample and relapse was 104 days (range 31–680). However, the median time from the highest chimerism value (%_max_) to relapse was 198 days and the median time from transplantation to %_max_ was 91 days (Table S1).

**TABLE 3:** Relapse prediction model based on maximum recipient chimerism (%_max_) in samples taken >30 days before relapse

Blood	All			Pediatric			Adult		
*Univariable*	*p‐*Value	OR	CI 95%	*P‐*Value	OR	CI 95%	*P‐*Value	OR	CI 95%
CD19	0.23	1.20	1.02–1.75	0.6	1.07	0.99–1.99	0.30	1.28	1.00–2.13
CD3	0.04	1.22	1.05–1.54	0.10	1.51	1.10–3.21	0.27	1.11	0.99–1.46
CD33	0.05	1.70	1.12–3.09	0.18	1.39	1.04–2.95	0.03	3.53	1.34–13.2
***Multivariable***									
CD3	0.03	1.25	1.06–1.60	0.07	1.63	1.12–3.81	‐	‐	‐
CD33	‐	‐	‐	‐	‐	‐	0.02	4.03	1.49–15.5
Patient age	0.16	0.97	0.93–1.03	0.75	1.04	0.81–1.37	0.05	0.94	0.87–1.00
MAC versus RIC	0.64	0.69	0.16–3.40	1.00	‐	‐	0.35	0.43	0.07–2.57
BM versus PB	0.05	4.7	1.13–27.1	0.09	4.84	0.81–41.6	0.93	1.14	0.03–40.0
ATG	0.54	0.68	0.19–2.36	0.37	3.67	0.28–173	0.54	0.60	0.12–3.32
**Bone Marrow**									
***Univariable***									
CD19	0.01	1.15	1.04–1.29	0.03	1.33	1.07–1.84	0.09	1.10	0.99‐1‐25
CD3	0.003	1.25	1.10–1.47	0.08	1.26	1.06–1.76	0.02	1.24	1.06–1.52
CD33	0.008	1.21	1.07–1.42	0.1	1.34	1.02–2.16	0.04	1.18	1.03–1.42
CD34	0.03	1.07	1.01–1.14	0.05	1.12	1.01–1.27	0.20	1.05	0.97–1.13
***Multivariable***									
CD3	0.004	1.24	1.09–1.46	0.05	1.27	1.07–1.75	0.02	1.26	1.05–1.57
Patient age	0.26	0.98	0.93‐1‐01	0.96	0.99	0.81‐1‐30	0.18	0.96	0.90–1.02
MAC versus RIC	0.99	1.01	0.24–4.84	1.00	‐	‐	0.87	0.88	0.18–4.56
BM versus PB	0.10	3.5	0.86–18.5	0.11	4.36	0.74–34.7	0.41	0.27	0.01–8.73
ATG	0.26	0.92	0.29–3.09	0.96	3.05	0.26–90.9	0.71	0.76	0.18–3.46

*Note*: Logistic regression analysis results are presented. Certain covariates in the pediatric subsets have been excluded due to few occurrences in the data set.

Abbreviations: ATG, antithymocyte globulin pretreatment; BM, bone marrow stem cell source; CI, confidence interval; MAC, myeloablative conditioning; *n*, number of patients; OR, odds ratio; PB, peripheral blood stem cell source; RIC, reduced intensity conditioning.

The same analysis was then performed using the chimerism values from blood samples. Despite having a larger number of chimerism results compared to bone marrow (501 vs. 225), univariable analysis demonstrated weaker associations between %_max_ in the three cell fractions and subsequent relapse. Analysis of CD3^+^ blood cells just passed the threshold for statistical significance and ROC‐analysis resulted in an inferior AUC of 0.68 compared to the bone marrow chimerism data. Setting the cutoff at 0.3% resulted in a sensitivity of 62% and a specificity of 64%.

**FIGURE 4:**
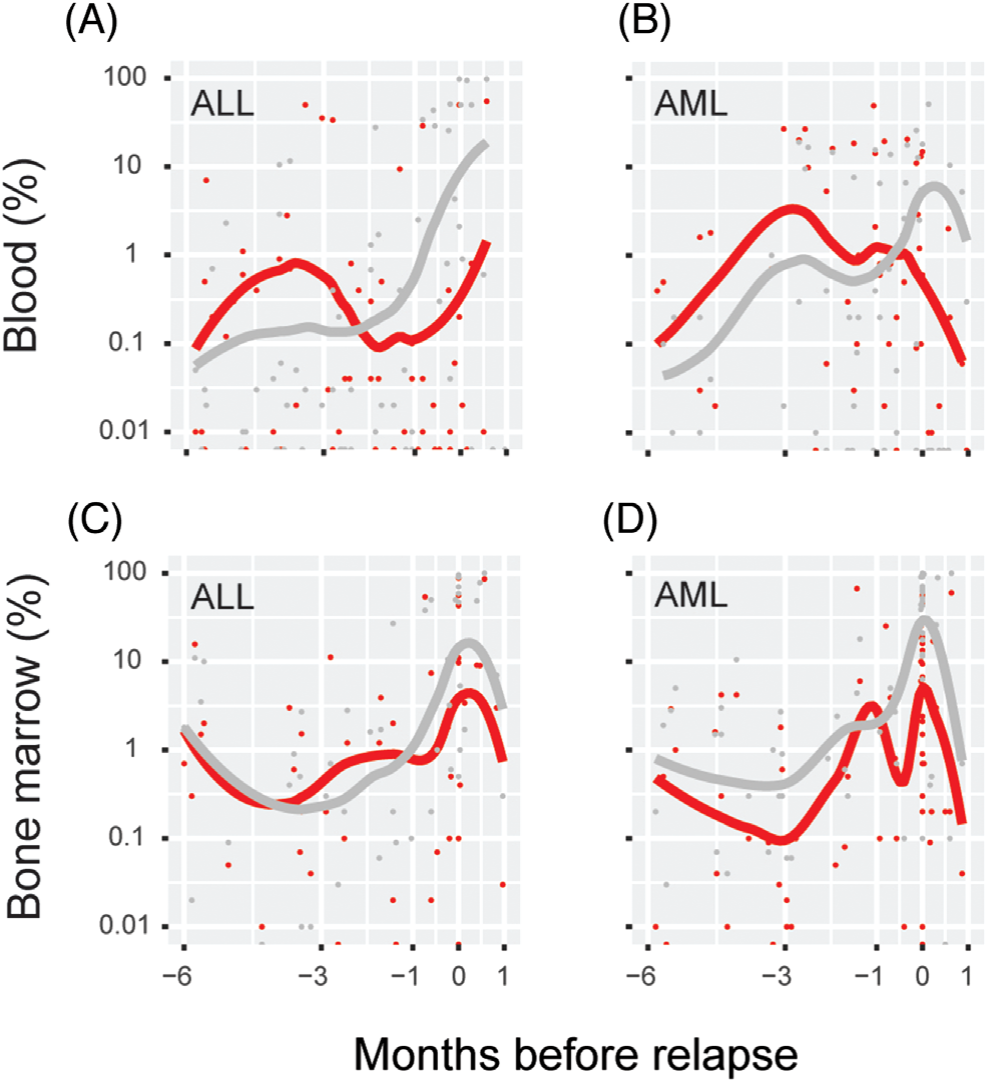
T cell and leukemia cell lineage chimerism kinetics during the months preceding a relapse. All chimerism results from hematopoietic stem cell transplantations (HSCTs) that resulted in a relapse in B cell acute lymphoblastic leukemia (ALL) (*n* = 28) and acute myeloid leukemia (AML) (*n* = 37) were extracted, and CD3^+^ T cell chimerism (red) was compared with the leukemia cell type, CD19^+^ B cell, or CD33^+^ myeloid cell chimerism for ALL and AML, respectively (gray). Blood samples (A and B) and bone marrow samples (C and D) are plotted separately, and a locally estimated scatterplot smoothing (LOESS) line was fitted to the data points for each cell type. The time of relapse for each HSCT has been aligned at 0 and the x‐axis

To visualize these changes in chimerism during the final months leading up to a relapse we plotted all available CD3^+^ cell and CD19^+^ cell chimerism values from relapsed HSCTs, and a line was fitted to each (Figure 4A,C). Notably, the numbers of samples contributed by each HSCT and consequently also influence on the fitted lines were not equal. This was done for blood and bone marrow samples separately. As expected, at the time of relapse there was a large proportion of recipient DNA in the CD19^+^ cell fraction of both sample types, presumably reflecting B‐ALL cells and thus acting as a marker of MRD. However, in both compartments relapse was preceded by a transient increase in CD3^+^ cell chimerism. To address if this is a general phenomenon observable also in other forms of leukemia, we retrieved similar data from our recently reported study of AML HSCTs [10] and constructed similar plots (Figure 4B,D). In this case, we compared the kinetics of chimerism results in CD3^+^ cells with cells positive for the myeloid cell marker CD33 and observed subtle signs the same phenomenon. Essentially, in the months preceding a relapse in B‐ALL or AML it is possible to discern a slight increase in T cell recipient chimerism before the more prominent increase in leukemia cell lineage chimerism becomes apparent at the time of relapse.

### 3.4 Prediction of relapse in pediatric versus adult HSCTs

Next, we hypothesized that the predictive performance would improve if pediatric and adult HSCTs were analyzed separately. In the adult age group, analysis of bone marrow samples gave similar results compared to the full data set. The predictive performance of %_max_ in CD3^+^ bone marrow cells was slightly better even, with a sensitivity of 73% and a specificity of 83%. After performing the same analysis in the pediatric age group only 34 HSCTs remained of which nine resulted in a relapse. Univariable analysis resulted in borderline *p*‐values for most sorted cell sample types. Interestingly, chimerism in CD3^+^ cells still appeared as a potential predictor of future relapse with a sensitivity of 63% and specificity of 88%, but the suggested optimal cutoff of 3.9% is distinctly higher than for adults (1.6%). No statistically significant association was observed for the blood chimerism data in the pediatric subset. In the adult age group, CD33^+^ cell chimerism seemed to be the most promising candidate in blood samples for the prediction of future relapse. Univariable analysis of %_max_ in blood CD33^+^ cells showed a significant association with relapse and setting the cutoff at 0.3% resulted in a sensitivity of 67% and a specificity of 73%.

### 3.5 Early complete donor chimerism in blood and relapse risk

**FIGURE 5:**
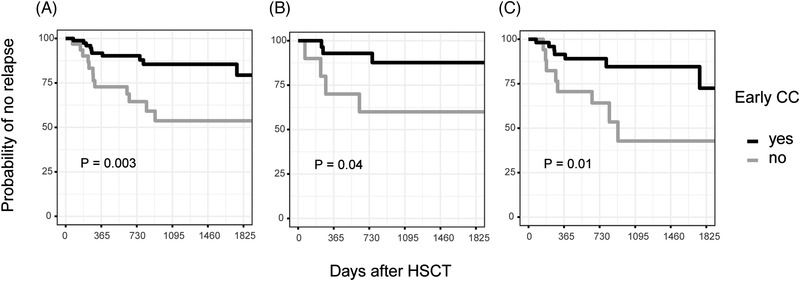
Complete chimerism in CD3^+^ cells early after hematopoietic stem cell transplantation (HSCT) **predicts lower relapse risk**. The lowest chimerism value in CD3^+^ cells from blood sampled during the first 60 days after HSCT was used to stratify patients based on if early complete chimerism was achieved or not. Kaplan–Meier plots depict the probability of not having a relapse as a function of time after HSCT depending on if early complete chimerism was achieved or not. The plots include data from (A) all patients (B) only pediatric patients and (C) only adult patients. The cutoff used to define complete chimerism for all patients was <0.3% in CD3^+^ cells but was adapted for the pediatric (<0.9%) and adult patients (<0.12%). *p*‐values were calculated using the log‐rank test. CC, complete chimerism

A complementary strategy for relapse prediction that also uses chimerism analysis is to risk‐stratify patients early after HSCT based on if complete chimerism (CC) is achieved or not. The rationale being that even slightly elevated chimerism results early after transplantation may suggest poor graft‐versus‐leukemia (GVL) effect and consequently an increased risk of subsequent relapse [15]. In a two‐step process we identified an optimal cutoff that we used to define CC, which varied depending on sample type and patient subset, and then modeled time to relapse based on if these criteria were met. Analogous to previous analyses we defined the variable %_min_ as the lowest chimerism value obtained during the first 60 days after HSCT. The analysis was limited to blood samples because bone marrow sampling during this time after HSCT for B‐ALL is infrequent at our center. HSCTs that resulted in a relapse within the first 60 days (*n* = 3) were removed from the analysis. Univariable and multivariable analysis using the full data set demonstrated a statistically significant association between %_min_ in CD3^+^ cells and subsequent relapse (Table S2). ROC‐curve analysis showed a moderate discriminative efficacy for %_min_ to predict relapse with an AUC of 0.69 (not shown). Youden's index suggested an optimal cutoff of 0.3%, which resulted in a sensitivity of 54% and a specificity of 80%. Next, we investigated if early CC using this low threshold of recipient DNA may be a useful means to stratify patients as having high or low risk of relapse. We dichotomized all patients based on if early CC was detected, defined as at least one peripheral blood chimerism value below 0.3% during the first 60 days after HSCT. Note that 121 HSCTs of which 22 resulted in a relapse were included. Early CC was observed in 45% of patients that later had a relapse and 80% of patients that did not have a relapse during the study time (χ2 *P* = 0.002). Univariable and multivariable analysis showed a statistically significant association between early CC and subsequent relapse (Table 4). Kaplan–Meier analysis showed a statistically significant lower probability of relapse in patients with early CC (log‐rank test *p* = 0.003) (Figure 5A). The probability of not having had a relapse 2 years after transplantation for patients that achieved early CC was estimated at 90% (95% CI 84–97) compared to 65% (49–85) (Table 4).

**TABLE 4:** Relapse prediction model based on detection of complete chimerism (CC) in blood samples taken the first 60 days after HSCT

	All			Pediatric			Adult		
*Logistic Regr*	*p‐Value*	OR	CI 95%	*p‐Value*	OR	CI 95%	*p‐Value*	OR	CI 95%
CD19	0.003	0.22	0.07–0.58	0.16	0.32	0.06–1.15	0.008	0.17	0.04–0.60
CD3	0.002	0.21	0.08–0.55	0.03	0.14	0.02–0.78	0.02	0.24	0.07–0.77
CD33	0.05	0.41	0.16–1.02	0.3	0.44	0.08–2.04	0.03	0.28	0.08–0.92
*Kaplan–Meier*	*P*	2‐yr	CI	*P*	2‐yr	CI	*P*	2‐yr	CI
CD19: CC	0.01	89	81–98	0.1	‐	‐	0.07	‐	‐
(MC)		(68)	(54–86)		‐	‐		‐	‐
CD3: CC	0.003	90	84–97	0.04	93	84–100	0.01	89	80–99
(MC)		(65)	(49–85)		(60)	(36–99)		(64)	(44–92)
CD33: CC	0.09	‐	‐	0.3	‐	‐	0.03	86	45–96
(MC)		‐	‐		‐	‐		(65)	(77–96)

*Note*: Logistic regression and Kaplan–Meier analysis results are presented. *p*‐Values for Kaplan–Meier analysis were calculated using the log‐rank test.

Abbreviations: 2‐yr, 2‐year probability (%) of not having had a relapse within the study time; CC, complete chimerism; CI, confidence interval; MC, mixed chimerism (defined as not having met the criterion for CC); OR, odds ratio.

CC was defined as: at least on recipient percentage value during the first 60 days after hematopoietic stem cell transplantation that was below the respective thresholds based on receiver operating characteristics analysis: (CD19, CD3, and CD33) 0.01, 0.3, and 0.06 for All; 0.2, 0.9, and 0.06 for Pediatric; and 0.01, 0.12, and 0.1 for Adult.

### 3.6 Relapse risk in pediatric versus adult HSCT

The same analysis was performed exclusively in the pediatric age group with no statistically significant associations between %_min_ and relapse. However, results from CD3^+^ cells appeared most promising (Table S2). The number of HSCTs available for this analysis was 45 of which seven resulted in a relapse. To discriminate between HSCTs that resulted in a relapse after the first 60 days or not ROC‐analysis (AUC = 0.73, not shown) suggested a cutoff of 0.9%, higher than for the full data set. Early CC was observed in 43% of HSCTs that later had a relapse and 84% that did not (χ2 *P* = 0.054). Univariable logistic regression showed a significant association between this dichotomous variable and relapse (*p* = 0.03, Table 4). Kaplan–Meier analysis showed a statistically significant lower probability of relapse also in this small subset of pediatric HSCTs when early CC, defined as at least one result <0.9% in CD3^+^ blood cells, was used for stratification (log‐rank test *p* = 0.04, Figure 5B). Similarly, analyzing the adult age group yielded no significant association between %_min_ and relapse. However, CD3^+^ cells appeared most promising (Table 4). Here, 76 HSCTs were available for analysis of which 15 resulted in a relapse. ROC‐curve analysis (AUC = 0.70, not shown) suggested a cutoff of 0.12% and using this value to define CC in the adult HSCTs a statistically significant association with relapse was observed. Early CC was confirmed in 47% of patients that later had a relapse and 79% of patients that did not have a relapse during the study time (χ2 *p* = 0.03). Kaplan–Meier analysis for the adult age group showed a statistically significant lower probability of relapse using this definition of early CC for stratification (log‐rank test *p* = 0.01) (Figure 5C).

## 4 DISCUSSION

We have previously reported that AML relapse post‐HSCT generally coincides with a prominent increase in recipient chimerism in cells positive for the myeloid marker CD33, essentially serving as a MRD marker [10]. However, given the sampling frequency of that retrospective material, single elevated chimerism values in any of the analyzed lineages (CD3, CD19, CD33, and CD34) did not consistently signal the upcoming relapse with time to intervene. Additionally, in the same study we demonstrated that achieving early CC, using the strict criteria of having at least one chimerism result <0.2% recipient in CD33^+^ blood or bone marrow cells during the first 60 days after HSCT, was associated with a lower risk of subsequent relapse. Based on these results we proposed that assessment of early CC could be useful for risk‐stratification and patients that fail to achieve these strict criteria of CC could benefit from more frequent monitoring, not just limited to chimerism.

Here, in the context of HSCTs for B‐ALL, we confirm the association between early CC and subsequent relapse. According to these data, patients that achieve early CC have a 10% risk of having a relapse within 2 months – 2 years post‐HSCT compared to 35% for those that do not. In our previous study of AML the corresponding results were 24% and 58% [10], reflecting the worse prognosis associated with this disease. Notably, the cutoff and cell lineage that we suggested as optimal to define CC differed from the AML study.

Moreover, we show that single chimerism values has potential to be used for B‐ALL relapse prediction at least 30 days before diagnosis. In our previous study of AML this was not the case [10]. Interestingly, relapse was not primarily associated with increased chimerism in the B cell lineage, as would be expected if it acted as a MRD marker, but rather in the T cell lineage. In figure 4, we have plotted chimerism data from HSCTs that resulted in a relapse and also included data from our previous study of AML [10]. The fitted lines suggest that there is often a transient increase in recipient T cell chimerism that precedes the more pronounced increase at the time of relapse of CD19^+^ and CD33^+^ chimerism, respectively. Furthermore, we examined at what timepoint the highest chimerism value in CD3^+^ bone marrow cells (excluding the first 30 days post‐HSCT and the last 30 days pre‐relapse) is detected, here defined as the variable %_max_ (Table S1). In this dataset, the increase in recipient chimerism that most robustly was associated with a subsequent relapse occurred relatively early after transplantation (median 91 days) and when further testing was performed it was not uncommon that much lower or negative values occurred before the relapse (not shown). Importantly, with the cutoffs suggested herein for relapse prediction in CD3^+^ bone marrow cells, chimerism results should primarily be used to rule in relapse (specificity 73%–88%) but not to rule it out (sensitivity 63%–67%).

It appears as if the events that ultimately lead to a relapse include cell type specific shifts in donor/recipient immune cell numbers that can be appreciated in blood and bone marrow several months before a manifest hematological relapse. One may speculate that this phenomenon reflects a subtle loss of graft integrity and/or graft rejection resulting in a loss of GVL‐effect and relapse, which has been suggested by others [14, 16, 17, 18]. An association between early mixed chimerism or increased chimerism in the T cell lineage and subsequent relapse has been reported for both AML and ALL [17, 19, 20, 21, 22]. In pediatric ALL, Preuner et al. similarly demonstrated that the reappearance of recipient DNA in CD8^+^ or CD34^+^ peripheral blood cells was associated with subsequent relapse [18]. Interestingly, they also observed that even when mixed chimerism later reverted to donor chimerism, the risk of relapse remained increased. This lends support to our strategy of making risk estimates based on single elevated chimerism values without considering what happens before or after.

This study was aimed at investigating how individual chimerism values may be interpreted for the purpose of predicting relapse and no comparison with the prognostic value of MRD was made. A recent comprehensive retrospective analysis compared chimerism in CD34^+^ cells with qPCR‐based MRD markers in ALL and concluded that the two methods were similarly effective and likely complement each other in predicting relapse [23].

Whether chimerism should be monitored in peripheral blood or bone marrow is often debated and is sometimes reduced to a question of sensitivity and specificity that only mandates adequate adjustment of cutoffs [9, 24]. If this is the case, sampling blood would naturally be preferable. The data we present herein does not contradict this idea but suggest that there may be additional qualitative difference in the information gained from the two compartments because bone marrow performed better than blood chimerism in our relapse prediction models despite fewer available values.

Whether relapse prediction in pediatric patients requires a higher chimerism cutoff compared to adults is less clear. There is a lack of studies that have compared results in children and adults and the striking differences in methodology and suggested cutoffs in published reports make comparisons between centers difficult [24].

In summary, it seems that chimerism can be more than a proxy marker for MRD. Using sensitive methods and adequate sampling frequency, chimerism may shed light on purely immunological events that take place before control of the malignant cells is lost, ultimately giving clinicians more time to intervene.

## AUTHOR CONTRIBUTIONS

*Conceptualization and design*: HL and DH. *Collection and assembly of data*: DV, SV, and HL. *Data analysis and manuscript writing*: HL. *Data interpretation and manuscript editing*: HL, DV, SV, MS, ATB, SM, and DH.

## CONFLICT OF INTEREST

Dan Hauzenberger is founder and co‐owner of Devyser AB. He is also part‐time employed by the company. Stephan Mielke has received speakers fee from Novartis and DNA Prime SA, has received travel support from and served in expert panel for Gilead/KITE, has received travel support and speakers fee from Celgene/BMS, has received research funding, travel support, and speakers fee from Kiadis Pharma, has served in expert panel for Bellicum, has received travel support and speakers fee from and has served in data safety monitoring board for Miltenyi, and has served in data safety monitoring board for Immunicum. The remaining authors have no conflict of interest to declare.

## ETHICS STATEMENT

This study was approved by the Swedish Ethical Review Authority and was conducted in accordance with the Declaration of Helsinki. Informed consent was obtained from all included patients.

## Supporting information



Supporting Material

## Data Availability

The data that support the findings of this study are available from the corresponding author upon reasonable request.
