# Supplementary material for: Early relapse prediction after allogeneic hematopoietic stem cell transplantation for acute lymphoblastic leukemia (ALL) using lineage‐specific chimerism analysis
Source: EJHaem. 2022 Sep 19;3(4):1277–86. doi: 10.1002/jha2.568 (PMC9713209; doi:10.1002/jha2.568)
Supplement: Supplementary file 1 — Supporting Material [file JHA2-3-1277-s001.docx]

**Supplementary data**

*Early relapse prediction after allogeneic hematopoietic stem cell transplantation for acute lymphoblastic leukemia (ALL) using lineage-specific chimerism analysis*

| **Table S1. Temporal relationship between highest CD3^+^ bone marrow chimerism value (%_max_) and relapse** | | | |
| --- | --- | --- | --- |
|  |  |  |  |
| *%_max_* | Days after HSCT | Days before relapse | Result |
| 0.01 | 210 | 442 | FN |
| 0.1 | 180 | 75 | FN |
| 0.2 | 98 | 1659 | FN |
| 0.3 | 35 | 175 | FN |
| 0.5 | 96 | 665 | FN |
| 0.6 | 76 | 108 | FN |
| 0.7 | 88 | 180 | FN |
| 0.8 | 208 | 31 | FN |
| 1.6 | 95 | 735 | TP |
| 1.7 | 86 | 213 | TP |
| 2.0 | 97 | 43 | TP |
| 2.3 | 118 | 680 | TP |
| 2.5 | 74 | 303 | TP |
| 3.0 | 64 | 111 | TP |
| 3.2 | 82 | 198 | TP |
| 3.9 | 180 | 51 | TP |
| 11.2 | 67 | 84 | TP |
| 12.8 | 90 | 828 | TP |
| 14.2 | 89 | 696 | TP |
| 15.7 | 91 | 173 | TP |
| 26.0 | 85 | 198 | TP |
| 38.4 | 274 | 355 | TP |
| The highest chimerism value from analysis of CD3^+^ bone marrow cell samples, excluding any samples taken the first 30 days after HSCT or the last 30 days before relapse, (termed %_max_) were used for a prediction model of subsequent relapse. Each row represents %_max_ for one individual and the number of days after HSCT and before relapse the sample was taken is indicated. The table includes both adult and pediatric patients and the suggested cutoff (1.6) is based on Receiver Operating Characteristics (ROC)-curve analysis of both age-groups combined. HSCT, hematopoietic stem cell transplantation; FN, false negative; TP, true positive. | | | |

| **Table S2. Relapse prediction model based on minimum recipient chimerism (%_min_) in blood samples taken the first 60 days after HSCT** | | | | | | | | | | | | |
| --- | --- | --- | --- | --- | --- | --- | --- | --- | --- | --- | --- | --- |
|  | | | | | | | | | | | | |
|  | **All** | | |  | | **Pediatric** | | |  | **Adult** | | |
| *univariable.* | *P* | OR | CI 95% |  | | *P* | OR | CI 95% |  | *P* | OR | CI 95% |
| CD19 | 0.09 | 1.05 | 1.00-1.13 |  | 0.13 | | 1.04 | 0.99-1.12 |  | 0.12 | 1.29 | 0.96-2.02 |
| CD3 | **0.006** | **2.74** | **1.36-6.02** |  | 0.17 | | 2.15 | 1.09-6.75 |  | 0.15 | 6.42 | 1.78-38.08 |
| CD33 | 0.41 | 1.33 | 0.61-2.71 |  | 0.56 | | 1.29 | 0.41-3.15 |  | 0.46 | 1.56 | 4.00-5.02 |
|  |  |  |  |  |  | |  |  |  |  |  |  |
| *multivariable* |  |  |  |  | |  |  |  |  |  |  |  |
| CD3 | 0.001 | 4.51 | 1.89-12.35 |  | | - | - | - |  | - | - | - |
| Patient age | 0.15 | 0.97 | 0.92-1.01 |  | | - | - | - |  | - | - | - |
| MAC vs RIC | 0.98 | 0.98 | 0.21-5.26 |  | | - | - | - |  | - | - | - |
| BM vs PB | 0.01 | 0.07 | 0.01-0.50 |  | | - | - | - |  | - | - | - |
| ATG | 0.54 | 1.48 | 0.40-5.23 |  | | - | - | - |  | - | - | - |
|  |  |  |  |  | |  |  |  |  |  |  |  |
| *ROC-curve* | AUC | YI | Sens/Spec |  | | AUC | YI | Sens/Spec |  | AUC | YI | Sens/Spec |
| CD19 | 0.68 | 0.01 | 68/68 |  | | 0.64 | 0.2 | 63/66 |  | 0.71 | 0.01 | 71/70 |
| CD3 | 0.69 | 0.3 | 54/80 |  | | 0.73 | 0.9 | 57/84 |  | 0.70 | 0.12 | 53/79 |
| CD33 | 0.63 | 0.06 | 50/71 |  | | 0.65 | 0.06 | 63/58 |  | 0.61 | 0.1 | 44/82 |
| Logistic regression and Receiver Operating Characteristics (ROC)-curve analysis results are presented. The lineage-specific chimerism data chosen for multivariable analysis is marked (bold). OR, odds ratio; CI, confidence interval; MAC, myeloablative conditioning; RIC, reduced intensity conditioning; Related vs not, HLA-identical sibling or haploidentical relative vs matched unrelated donor; BM, bone marrow stem cell source; PB, peripheral blood stem cell source; AUC, area under the curve; YI, Youden’s index; Sens, sensitivity; Spec, specificity. | | | | | | | | | | | | |
